# New-type urbanization and rural revitalization: A study on the coupled development of the Yangtze River Economic Belt, China

**DOI:** 10.1371/journal.pone.0314724

**Published:** 2025-01-03

**Authors:** Yan Wang, Ling Wang

**Affiliations:** Institute For Chengdu-Chongqing Economic Zone Development, Chongqing Technology and Business University, Chongqing, China; Aalto University, FINLAND

## Abstract

The coupled development of new-type urbanization (NTU) and rural revitalization (RR) represents a critical proposition put forth by China for forging a novel paradigm of urban-rural relationship. Initially, this study employs the entropy method to quantify NTU and RR. Subsequently, it carries out a comprehensive analysis concerning their coupled relationship with the relative development degree model (RDDM), coupled coordination degree model (CCDM), Dagum Gini coefficient, kernel density estimation, and Tobit model. The findings drawn from the study indicate from 2011 to 2022, NTU and RR in the Yangtze River Economic Belt (YREB) have exhibited a consistent upward trajectory, but lagging NTU disorders are widely distributed and numerous. The coupled coordination degree (CCD) of NTU and RR constantly improves, transitioning from moderate imbalance to primary coordination, exhibiting a spatial distribution of a "high in the east and low in the west". The relative disparity between the coupled development of NTU and RR demonstrates a slowly narrowing trend, whereas the absolute disparity indicates an expanding trend. Among the influencing factors, the development of the agricultural industry exerts the most significant positive impact on the coupled development, whereas the level of financial support for agriculture exerts a dampening effect, which is heterogeneous in nature.

## 1. Introduction

The 2022 UN-Habitat publication, World Cities Report: Envisioning the Urban Future, observes that global urbanization will persist, with rates projected to reach 68% by 2050, up from 56% in 2021. This increase will result in 2.2 billion new urban residents, primarily situated in Africa and Asia. China, a significant presence in Asia, has experienced the world’s largest and rapid urbanization. Its urbanization rate has climbed from 17.92% in 1978 to 65.2% in 2022, contributing to a widening income disparity between its urban and rural populations and generating a dual economic strain: urban prosperity coupled with rural decline [1]. In response, the Chinese government has advanced the implementation of new-type urbanization (NTU) and rural revitalization (RR) strategies, emphasizing their function as a “double-wheel drive” for development. The Yangtze River Economic Belt (YREB), pivotal to China’s overall advancement, serves as the pricipal region for promoting the NTU and RR strategy. IHowever, it faces the concurrent challenges of rapid urbanization and an escalating urban-rural income disparity. Consequently, achieving coupled NTU and RR development in the YREB entails significant complexities. Several critical questions arise: What is the current level of coupled development of NTU and RR in the YREB? What are the spatial and temporal patterns, regional disparity, and dynamic evolutionary characteristics of this coupled development? Which factors affect the coordinated development of the two strategies? How can their positive interaction be promoted specific to local conditions? Answers to these questions hold significant theoretical value and practical implications.

The concept of NTU in China originates from a recognition of the factors hindering conventional urbanization. In contrast to its predecessor, NTU attaches greater importance to a human-centric approach [2], and pays more attention to green and sustainable development [3]. There is also a greater emphasis on the city as a complex system composed of many elements, implying that the index system should be constructed from the dimensions of population, population, economy, land, society, ecology [4–6], and that comprehensive studies should be carried out at different geographical scales, from the national level down to city clusters, watersheds, provinces, cities, and counties [7–12]. The YREB is a typical region representing different stages of China’s urbanization and industrialization [13]. Exploring its development status, progression, spatial and temporal characteristics, spatial clustering pattern and influencing factors of NTU in the YREB [14–16], which can help to promote the the successful implementation of NTU. However, it still experiences general problems in the development process of urbanization. These include land resource allocation disparities, unidirectional population flows, barriers to industrial structure advancement, environmental degradation, urban-rural imbalances, and inadequate rural development [17–19], leading to the obstruction of the construction process of NTU.

During the 1970s, the accelerated expansion of urban economies and the increasing disparity between urban and rural areas in numerous countries prompted literature critique of the "urban tendency" theory. This period saw a surge in recognition of the importance of rural development, as well as prompting national governments to explore the rural revitalization. Notable examples include the UK’s Rural Development Plan [20], Japan’s One Village One Product Movement [21], Korea’s New Village Movement [22], Australia’s Two-Track Rural Development Strategy [23], and Switzerland’s Regional Nature Park Project [24]. China, meanwhile, has implemented its RR strategy. This strategy centers around five key facets: "prosperous industry, ecological livability, civilized countryside, effective governance, and affluent life" [25]. As a result, a sophisticated evaluation index system has been established to measure the progress of China’s RR [26–28]. And researchers have employed methods such as entropy, Dagum’s Gini coefficient, and Kernel Density Estimation to offer an in-depth analysis of its current state of development, regional disparity, and spatial and temporal evolution [29–31].

The coupled development of NTU and RR is rooted in China’s specific national conditions. NTU and RR exist in a coupled relationship, represented by mutual complementation and promotion [32]. NTU facilitates RR, while the RR strategy offers a significant avenue for addressing challenges encountered during the advancement of NTU, such as the "big city disease" [33]. These two strategies share common values, goal convergence, subject consistency, and policy integration [34, 35]. However, a number of existing challenges presents significant obstacles. These challenges include the instability of employment and income, the challenge of establishing robust pillar industries, inconsistencies in public opinion, limitations in governmental capacity, the inadequate empowerment of farmers as primary stakeholders, the underdeveloped state of the rural factor market, the persistent challenge of bridging the urban-rural disparity, and the need for a more effective eco-compensation mechanism for rural ecological restoration. These factors collectively hinder the coupled development of NTU and RR [36–38]. Therefore, a more comprehensive understanding of the interactions between these two systems is crucial to cultivating their coordinated advancement.

Relevant studies on NTU, RR and the coupled development of NTU and RR offers a valuable foundation and for further analysis. Research indicates a consensus among researchers and practitioners on the importance of accelerating the coupled development of NTU and RR. In addition, as regions with high levels of urbanization progressively expand inland areas along the Yangtze River Basin and the Yellow River Basin, promoting coupled development in the YREB has become increasingly necessary, and this need has been acknowledged by both scholars and practitioner. However, existing studies often exhibit limitations in terms of their systematic and comprehensive nature. Many analyses focus on specific aspects of NTU or RR, neglecting to fully address their intersections. Moreover, while the significance of the YREB is recognized, in-depth analyses of this region remain limited. Current research primarily evaluates these dynamics from a provincial perspective, which poses challenges in capturing the characteristics of coupled development at a more in-depth level. To gain a more detailed and accurate understanding, it is essential to appraise regional disparity, spatial and temporal evolution and influencing factors with prefecture-level cities as the research object.

**Fig 1:**
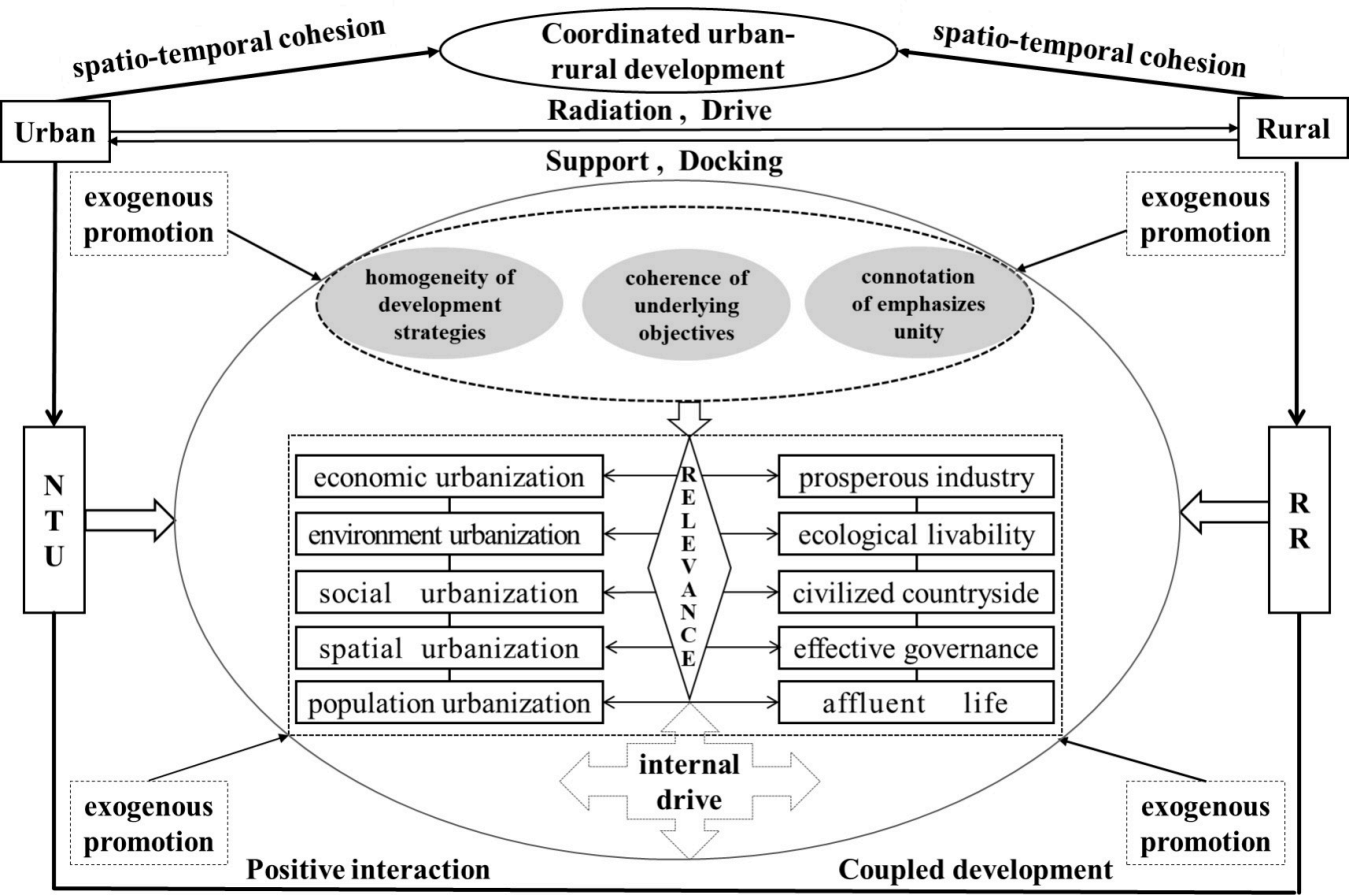
Theoretical framework of this paper.

Therefore, this study focuses on the YREB, constructing a theoretical framework for the coupled development of NTU and RR. A comprehensive evaluation index system is designed to appraise the patterns, regional disparity, spatio-temporal evolution, and influencing factors driving coupled development. Based on these findings, the study proposes targeted countermeasures and suggestions to promote the coupled development of NTU and RR in the YREB. The theoretical framework is illustrated in Fig 1.

## 2. Methods and materials

### 2.1 Study area and data sources

**Fig 2:**
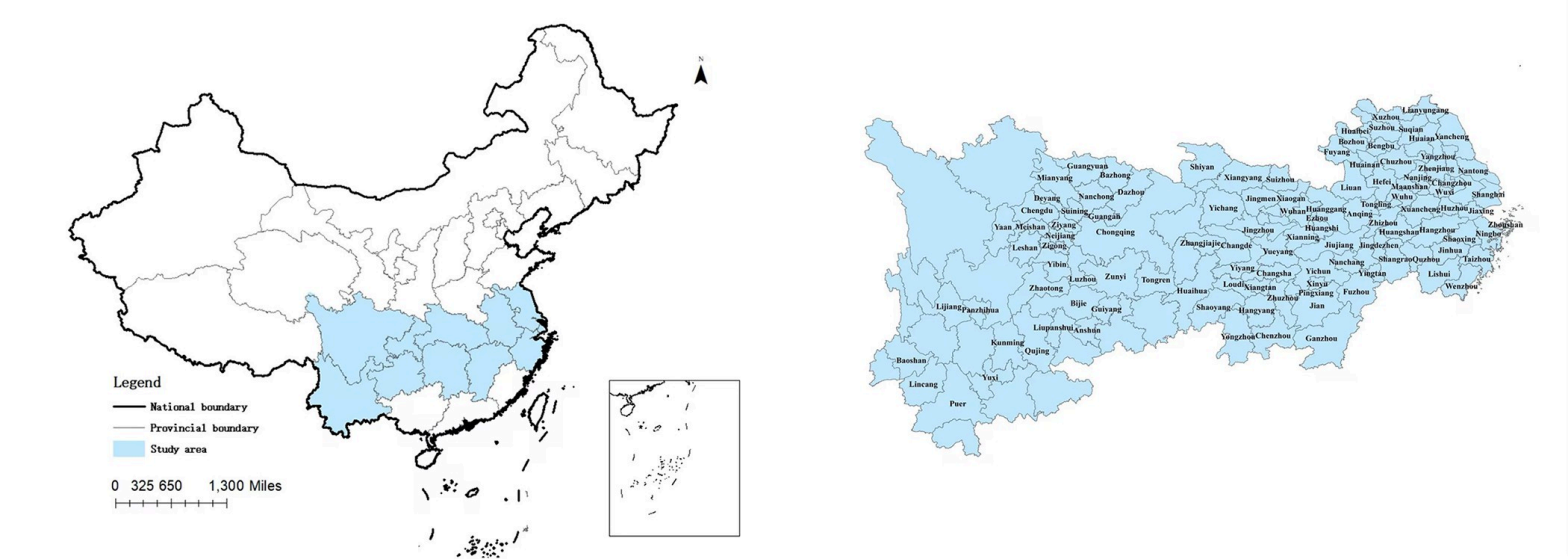
Geographical location of the study area.

Regions with high levels of urbanization are gradually expanding to inland areas along the Yangtze River and Yellow River basins. However, research on NTU and RR has paid little attention to the YREB. Therefore, this study focuses on the YREB as its research object. Including three major regions in China, the YREB covers 11 provinces (municipalities) with an area of approximately 2.0523 million square kilometers, representing 21.4% of the national total. Considering data availability, our research sample comprises 110 prefecture-level cities in the 11 provinces (municipalities) of the YREB (Fig 2), categorized into three regions: upstream, midstream, and downstream. The downstream region (DR) includes Shanghai, Jiangsu, Zhejiang, and Anhui; the midstream region (MR) includes Jiangxi, Hubei, and Hunan; and the upstream region (UR) includes Chongqing, Sichuan, Guizhou, and Yunnan.

This article employs a sample interval of 2011–2022 and constructs panel data for 110 prefecture-level cities. The research data primarily originates from the China Urban Statistical Yearbook, China Urban Rural Construction Statistical Yearbook, various urban statistical yearbooks, and the National Economic and Social Development Statistical Bulletin. Data on the number of national key leading enterprises in agricultural industrialization were compiled from the list published by the Ministry of Agriculture and Rural Affairs of the People’s Republic of China (https://www.moa.gov.cn), with linear interpolation utilized to address a small number of missing values.

### 2.2 Indicator selection

#### 2.2.1 New-type urbanization

The "National New-type Urbanization Plan (2021–2025)" clearly proposes accelerating population urbanization, promoting the construction of new-type cities, and cultivating the integration of urban and rural development. The plan also outlines specific requirements for various aspects, including population, economy, society, and environment. Therefore, by synthesizing the development of NTU in the YREB with prior research [39, 40], this article establishes an NTU evaluation index system based on five dimensions: population urbanization, economic urbanization, social urbanization, environment urbanization, and spatial urbanization (Table 1).

**Table 1:** The evaluation index system of NTU.

Primary indicators	Secondary indicators and unit	Attribute
Population urbanization	X_1_ Urban registered unemployment rate(%)	-
	X_2_ Urban population density(person/km^2^)	+
	X_3_ Urbanization rate(%)	+
Economic urbanization	X_4_ Per capita disposable income of urban residents(Yuan)	+
	X_5_ The proportion of output value of secondary and tertiary industries in GDP(%)	+
	X_6_ GDP per capita(Yuan)	+
Social urbanization	X_7_ Number of people participating in urban basic pension insurance at the end of the year(Person)	+
	X_8_ Share of education spending in fiscal spending(%)	+
	X_9_ Number of buses per 10,000 people(Vehicle)	+
Environment urbanization	X_10_ Green space rate of built-up district(%)	+
	X_11_ Comprehensive utilization rate of industrial solid waste(%)	+
	X_12_ Urban sewage centralized treatment rate(%)	+
Spatial urbanization	X_13_ Per capita paved road are(m^2^)	+
	X_14_ GDP per land(100 million Yuan/square kilometer)	+
	X_15_ Urban built-up area(Square kilometer)	+

#### 2.2.2 Rural revitalization

The "Strategic Plan for Rural Revitalization (2018–2022)" clearly identifies the advancement of rural industries, talent cultivation, cultural enrichment, ecological conservation, and robust organizational structures as key areas for revitalization. Therefore, Chinese academia has formed a relatively comprehensive evaluation index system for RR. Drawing upon the strategic blueprint of China’s RR plan and existing research [41], this article presents a RR evaluation index system structured around five key pillars: prosperous industry, ecological livability, civilized countryside, effective governance, and affluent life (Table 2).

**Table 2:** The evaluation index system of RR.

Primary indicators	Secondary indicators and unit	Attribute
Prosperous industry	Y_1_ Total grain output (10000 tons)	+
	Y_2_ Number of National Key Leading Enterprises in Agricultural Industrialization (Individual)	+
	Y_3_ The ratio of the total output value of agriculture, forestry, animal husbandry and fishery to the number of employed people in the primary industry (10000 Yuan/people)	+
Ecological livability	Y_4_ Comprehensive utilization rate of livestock and poultry waste (%)	+
	Y_5_ Amount of fertilizers (10000 tons)	-
	Y_6_ Rural greening rate (%)	+
Civilized countryside	Y_7_ Percentage of full-time teachers in rural compulsory education schools with bachelor’s degree or above (%)	+
	Y_8_ Percentage of rural residents’ expenditure on education, culture and entertainment (%)	+
	Y_9_ Number of cultural stations in rural areas (Individual)	+
Effective governance	Y_10_ The proportion of village directors and secretaries shouldering responsibilities (%)	+
	Y_11_ Percentage of administrative villages that have prepared village plans (%)	+
	Y_12_ Percentage of administrative villages that have carried out village improvement (%)	+
Affluent life	Y_13_ Urban-rural income ratio (%)	-
	Y_14_ Per capita income in rural areas (Yuan)	+
	Y_15_ Engel coefficient of rural residents (%)	-

#### 2.2.3 Influencing factors

Considering the coupled development of NTU and RR in the YREB, influenced by external factors, this study incorporates the following indicators, as identified in related studies [42]: ① Opening up level (OPL): Reflected by the actual amount of foreign investment utilized, attracting foreign direct investment is a practical avenue for promoting both NTU and RR, representing a significant driver for their coupled development. ② Transportation level (TL): Highway mileage was chosen as the measure. Highway transportation is a pioneering and service facility for advancing both NTU and RR, and fundamental to their coupled development. ③ Technological innovation level (TIL): The number of authorized patent applications is selected. Improvements in the technological innovation level are fundamental to enhancing the innovation capacity of both NTU and RR, acting as a driving force for their coupled development. ④ Financial support for agriculture level (FSAL): Measured by the proportion of agricultural, forestry, and water affairs expenditure. Financial support for agriculture plays a guiding and regulatory role in the development of NTU and RR, representing a guarantor for their coupled development. ⑤ Agricultural mechanization level (AML): The per capita total power of agricultural machinery has been chosen to express this. Promoting agricultural mechanization is a crucial fulcrum for advancing both NTU and RR, representing a key force in their coupled development. ⑥ Agricultural industry development (AID): Represented by the main business income of large-scale agricultural product enterprises, and the agricultural product processing industry is a critical lever for promoting NTU and RR, acting as a supporting force for their coupled development. Table 3 offers the variable settings for the influencing factors of coupled coordination degree (CCD) and the descriptive statistical results for each variable.

**Table 3:** Variable setting and descriptive statistical results of factors influencing CCD.

Variable category	Variable name	Variable symbol	Mean	Standand detiation	Min	Max
Explained variable	CCD	D	0.314	0.056	0.171	0.606
Explanatory variable	Opening up level	OPL	12.370	1.752	6.180	16.510
	Transportation level	TL	9.518	0.601	7.181	12.120
	Technological innovation level	TIL	8.036	1.561	3.555	12.350
	Fiscal support of agricuture level	FSAL	2.455	0.396	1.189	4.310
	Agricultural mechanization level	AML	9.391	0.420	7.648	10.360
	Agricultural industry development	AID	7.848	0.395	6.336	8.809

### 2.3 Methodology

#### 2.3.1 Entropy model

The entropy method was initially proposed by the German physicist Rudolf Clausius in 1865, subsequently introduced by Claude Shannon into the information theory, and gradually developed to be applied to the evaluation of complex systems with many indicators and high correlation. The entropy method can significantly mitigate the inaccuracy caused by human subjective factors (Long et al., 2022) [43] and enhance the objectivity of weight determination (Gisleine et al., 2022) [44]. Therefore, this article employs the entropy method to compute an index for both NTU and RR. Firstly, the threshold method is utilized to standardize the original data, and the standardized data is shifted by 0.00001 units to facilitate calculations. Secondly, the weight (*P*_*it*,*j*_) of the j-th indicator for the i-th city in year t is measured. Thirdly, the information entropy (*E*_*j*_) and redundancy (*D*_*j*_) of the j-th indicator are calculated. Fourthly, the weight (*W*_*j*_) of the j-th indicator based on information entropy and redundancy is computed. Fifthly, the weights are utilized to obtain the NTU index (*S*_*it*_) and RR index (*U*_*it*_) of the i-th city in year t. The specific calculation formula is:

$$Positive\;indicators:{\;Y}_{it,j}=\frac{X_{it,j}-min\{X_{it,j}\}}{\max\left\{X_{it,j}\right\}-min\{X_{it,j}\}}+0.00001$$ (1)

$$Negative\;indicators:{\;Y}_{it,j}=\frac{max\{X_{it,j}\}-X_{it,j}}{\max\left\{X_{it,j}\right\}-min\{X_{it,j}\}}+0.00001$$ (2)

$$P_{it,j}=Y_{it,j}/\sum_{i=1}^{N}\sum_{t=1}^{T}Y_{it,j}$$ (3)

$$E_{j}=-\frac{1}{lnT}\sum_{t=1}^{T}P_{it,j}\times ln(P_{it,j})$$ (4)

$$D_{j}=1-E_{j}$$ (5)

$$W_{j}=\frac{D_{j}}{\sum_{j=1}^{m}D_{j}}$$ (6)

$$S_{it}=\sum_{j=1}^{m}W_{j}\times Y_{it,j}$$ (7)

$$U_{it}=\sum_{j=1}^{m}W_{j}\times Y_{it,j}$$ (8)

where *Y*_*it*,*j*_ represents the dimensionless variable of the j-th indicator of the i-th city in year t. *min* {*X*_*it*,*j*_} and *max* {*X*_*it*,*j*_} denotes the threshold, *min* {*X*_*it*,*j*_} which is the minimum value in the indicator, *max* {*X*_*it*,*j*_} depicts the maximum value in the indicator, *N = 110* indicates the number of cities, *T = 12* symbolizes the number of years, and *m* expresses the number of evaluation indicators.

#### 2.3.2 Relative development degree model (RDDM)

To express the relative development degree (RDD) of the two systems of NTU and RR, this paper introduces the RDDM, the model expression is:

$$R=\frac{S}{U}$$ (9)

where *R* represents the RDD, *S* and *U* denote the index of NTU and RR, respectively. The classification criteria for the R-value refer to previous studies [45], there are three types: lagging dysfunction of NTU (0<R≤0.8), NTU and RR co-limiting disorders (0.8<R≤1.2), lagging dysfunction of RR (R>1.2).

#### 2.3.3 Coupled coordination degree model (CCDM)

Coupled relationships are ubiquitous, and the degree of coupled can be employed to evaluate the coupled relationship between two systems. However, significant differences in the developmental levels of two different systems can lead to "inefficient coupling" and "efficient coupling" when calculating the coupled degree. Therefore, this paper utilizes the CCDM to explore the coupled relationship between NTU and RR in the YREB. The calculation steps of the model are as follows:

$$C=\frac{\sqrt{S\times U}}{S+U}$$ (10)

T=αS+βU (11)

$$D=\sqrt{C\times T}$$ (12)

where *C* represents the coupled degree of NTU and RR, *S* and *U* denote the NTU and RR index, respectively. T depicts the combined coordination index of the two systems, *α* and *β* indicate the coefficient to be determined, *α* = *β* = 0.5. D symbolizes the CCD between NTU and RR, the value range is [0, 1], the larger the value, the better the coupling between the two. Refer to related studies [46], this paper classified the CCD is divided into five stages: high coordination (0.8<D≤1), moderate coordination (0.6<D≤0.8), low coordination (0.4<D≤0.6), moderate imbalance (0.2<D≤0.4), and severe imbalance (0<D≤0.2).

#### 2.3.4 Dagum Gini coefficient

This paper employs the Dagum Gini coefficient to appraise the presence of regional disparities in the coupled development between NTU and RR. The Dagum Gini coefficient is able to decompose the overall Gini coefficient (*G*) into intra-regional disparities (*Gw*), inter-regional disparities (*Gnb*), and hypervariable density (*Gt*). The calculation method is as follows:

$$G=\frac{\sum_{j=1}^{k}\sum_{h=1}^{k}\sum_{i=1}^{n_{j}}\sum_{r=1}^{n_{h}}\left|y_{ji}-y_{hr}\right|}{2n^{2}\bar{y}}$$ (13)

$$G_{w}=\sum_{j=1}^{k}G_{jj}p_{j}s_{h}$$ (14)

$$G_{nb}=\sum_{j=2}^{k}\sum_{h=1}^{j-1}G_{jh}(p_{j}s_{h}+p_{h}s_{j})D_{jh}$$ (15)

$$G_{t}=\sum_{j=2}^{k}\sum_{h=1}^{j-1}G_{jh}(p_{j}s_{h}+p_{h}s_{j}){(1-D}_{jh})$$ (16)

$$G=G_{w}+G_{nb}+G_{t}$$ (17)

where *y*_*ji*_ (*y*_*hr*_) denotes the CCD of NTU and RR of city *i(r)* in the *j(h)* region, *n* depicts the total number of all cities, *n*_*j*_ (*n*_*h*_) represents the number of cities in the *j(h)* region, and *K = 3* connotes the number of dividing regions, which $\bar{y}$ implies the average value of the CCD of all cities.

#### 2.3.5 Kernel density estimation

Based on the analysis of regional relative disparities, Kernel density estimation helps identify absolute disparities in the CCD between NTU and RR, which in turn, reflects the dynamic evolution of the CCD. Assuming that *f*(*x*) is the density function of the CCD between NTU and RR, the formula is expressed as:

$$f(x)=\frac{1}{Nh}\sum_{i=1}^{N}K(\frac{X_{i}-\overset{-}{x}}{h})$$ (18)

$$K(x)=\frac{1}{\sqrt{2\pi }}exp(-\frac{x^{2}}{2})$$ (19)

where *N* represents the total number of observations, *X*_*i*_ denotes the observation with independent and same distribution characteristics, $\overset{-}{x}$ indicates the mean value of all observations, *K*(*x*) depicts the kernel density function, *h* symbolizes the bandwidth, if the bandwidth is bigger, the density function image is smoother, which indicates that the estimation accuracy is lower. In this paper, we choose Gaussian kernel function to estimate the dynamic evolution of the CCD between NTU and RR.

#### 2.3.6 Tobit model

When selecting Tobit models, conditional maximum likelihood estimation is not feasible for the fixed-effects Tobit model as sufficient statistics for individual heterogeneity cannot be found. Generally, only the mixed Tobit model and the random-effects Tobit model are considered. In this paper, the LR test indicates that the random effects model is superior to the mixed Tobit model. Therefore, the random effects Tobit model is employed for interpretation. The model is constructed as follows:

$$D_{it}=\gamma {lnX}_{it}+u_{i}+\varepsilon_{it}$$ (20)

where *D*_*it*_ denotes the measured CCD between NTU and RR, X_it_ represents each influencing factor, *γ* depicts the influence coefficient, which is the most important observation, *u*_*i*_ indicates the individual effect, and *ε*_*it*_ symbolizes the random error term.

## 3. Results

### 3.1 Spatio-temporal changes in the level of NTU and RR

**Fig 3:**
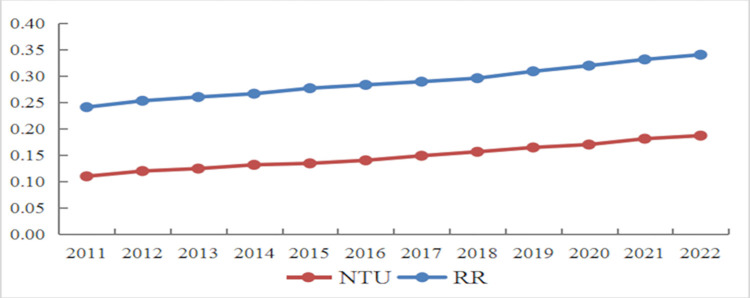
Changes of NTU and RR indexes from 2011 to 2022.

The entropy method was employed to measure the development indices of NTU and RR for 110 cities in the YREB between 2011 and 2022. Development indices for each city across all years were then averaged to determine the overall level of NTU and RR in the YREB (Fig 3). Calculated from the overall mean value, the NTU index increased from 0.1101 in 2011 to 0.1872 in 2022, demonstrating an average annual growth rate of 4.96%. Similarly, the RR index rose from 0.2411 in 2011 to 0.3404 in 2022, with an average annual growth rate of 3.64%. The development levels of both NTU and RR exhibit a consistent, upward trend year by year. However, the RR index consistently surpasses the NTU index in the same period, and both indices remain relatively low overall.

**Fig 4:**
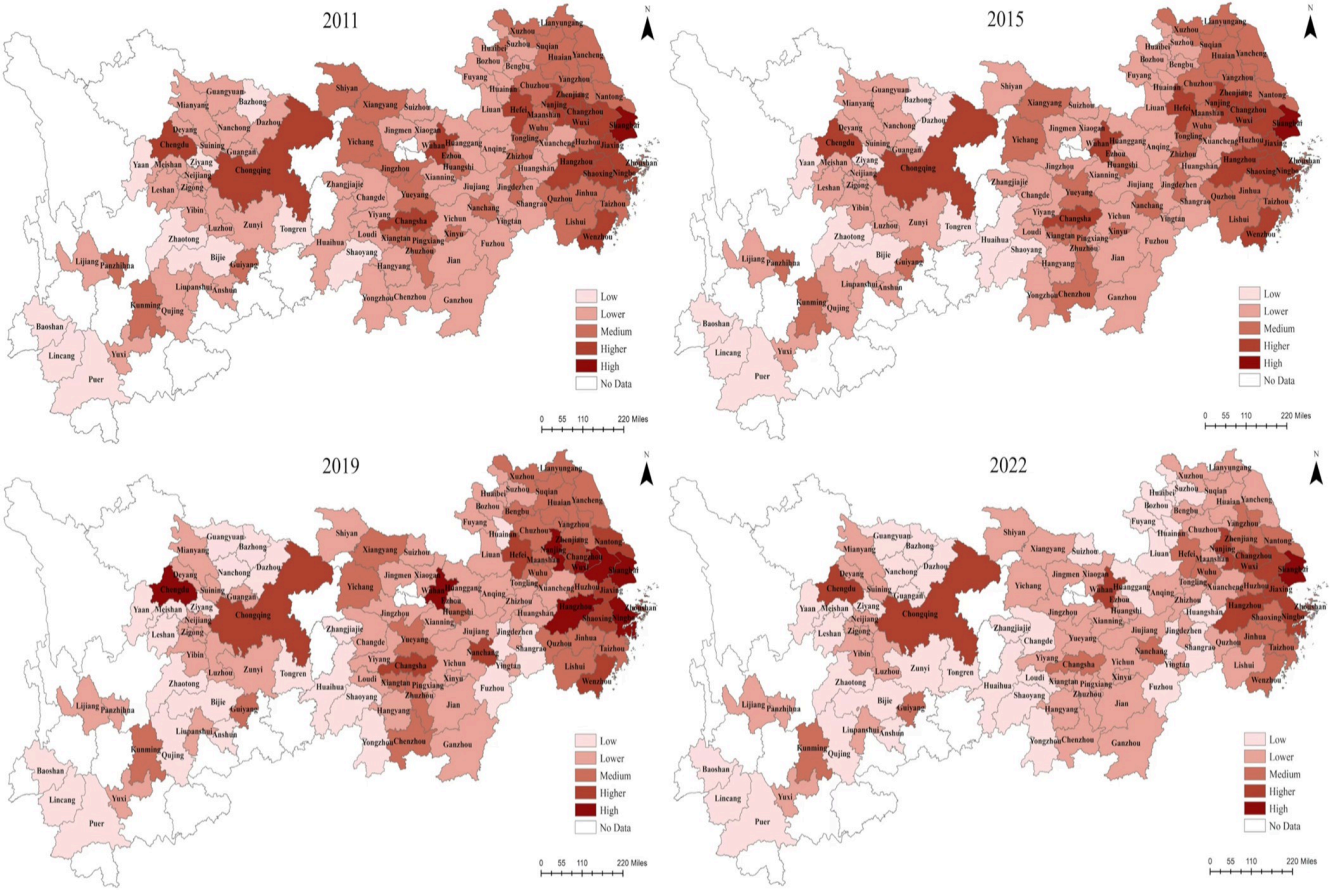
Spatial evolution of NTU in the YREB, 2011, 2015, 2019, 2022.

**Fig 5:**
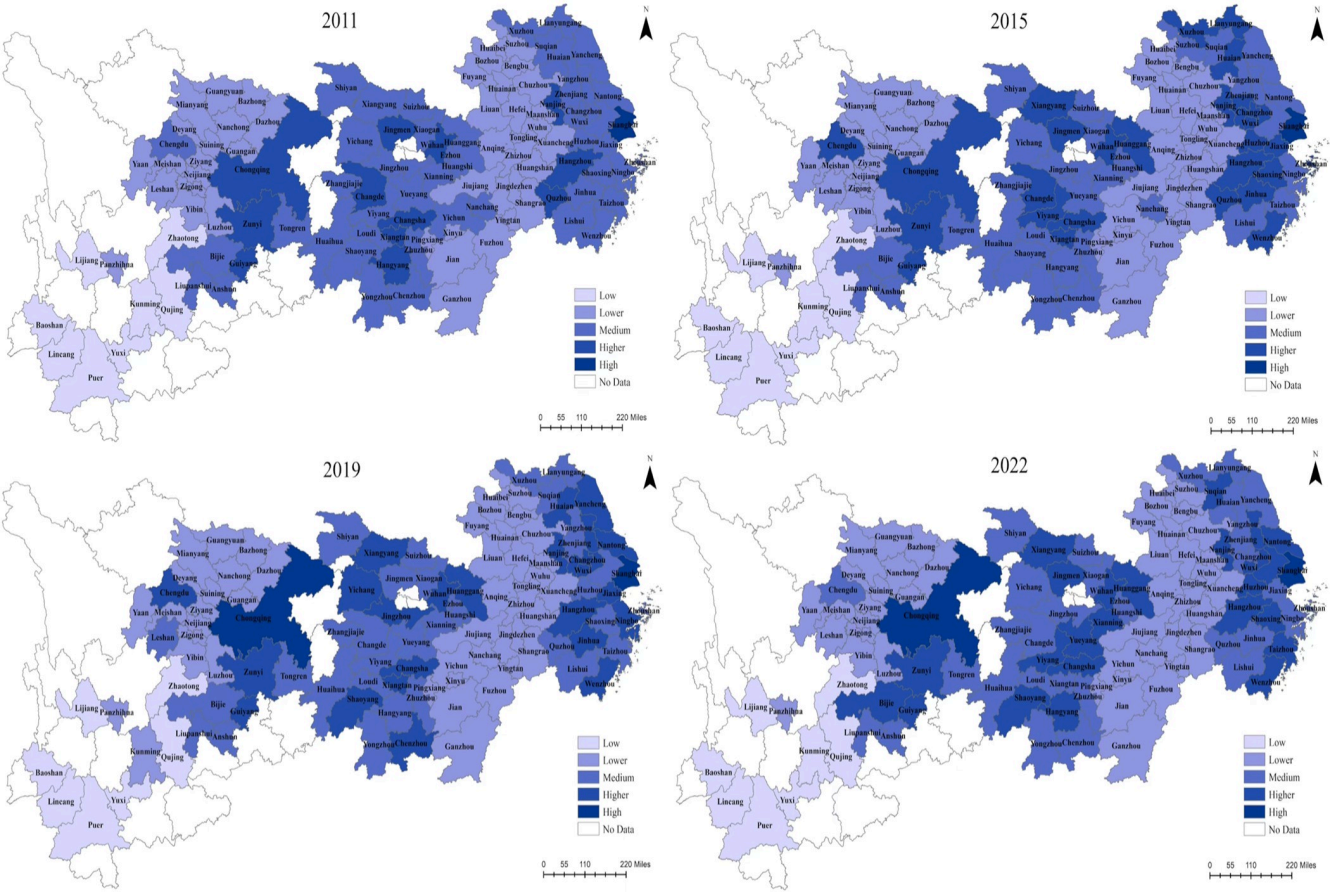
Spatial evolution of RR in the YREB, 2011, 2015, 2019, 2022.

To better illustrate the development of NTU and RR in the YREB throughout the study period, four time nodes (2011, 2015, 2019, and 2022) were selected, and the indices were categorized into five types (High, Higher, Medium, Lower, and Low) utilizing the natural break points method in ArcGIS 10.8 (Figs 4 and 5). The NTU and RR index of each region of the YREB have significant spatial variability from 2011 to 2022. The Yangtze River Delta Urban Agglomerations (YRDUA), centered around Shanghai, represent a concentrated area of high values, whereas the UR is reflected by a concentration of low values. This pattern illustrates a spatial pattern of decreasing levels of development from DR to UR. In summary, while the series of initiatives undertaken by the YREB to advance NTU and RR have begun to show progress, regional disparities persist.

### 3.2 The RDD between NTU and RR

**Fig 6:**
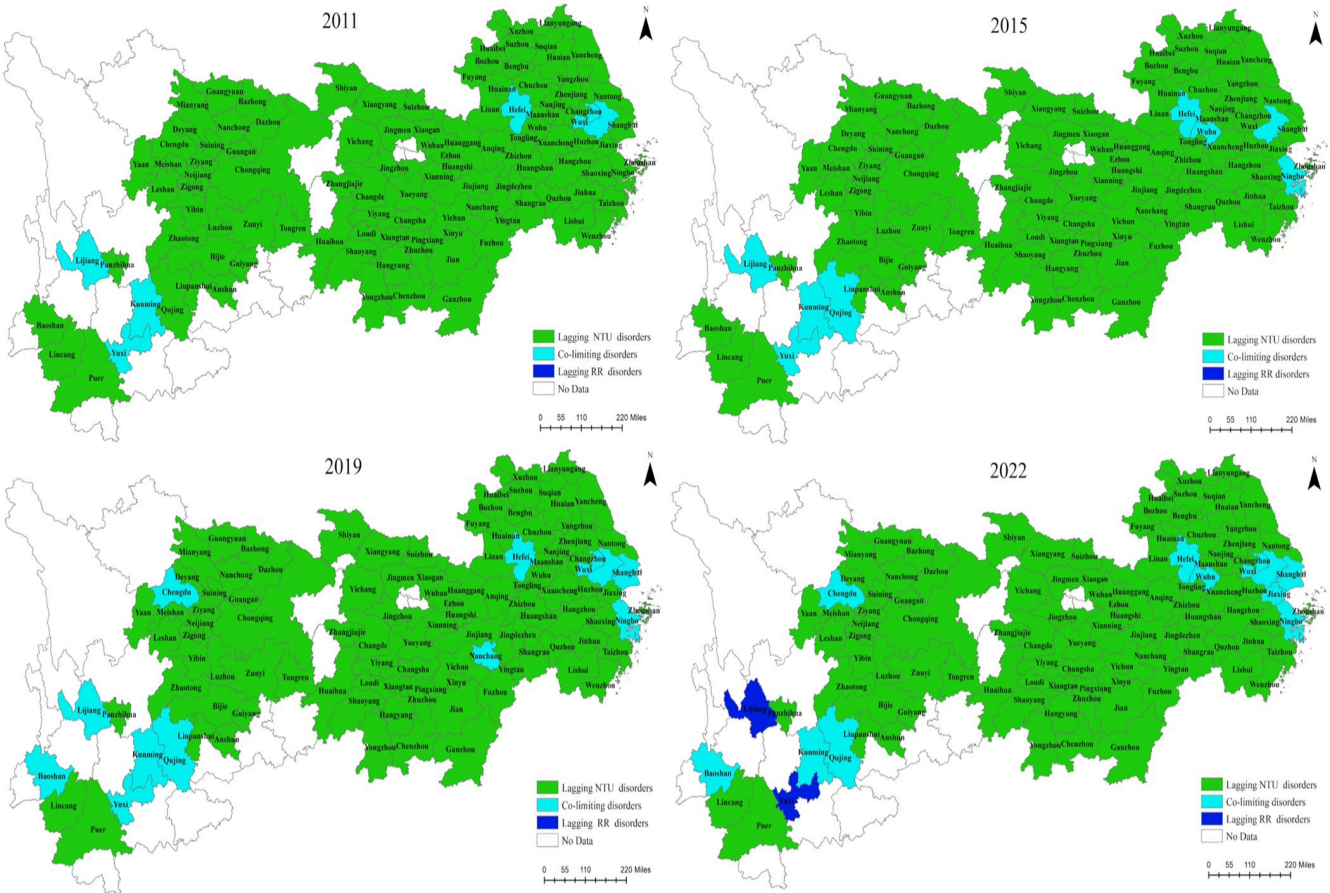
The RDD between NTU and RR, 2011, 2015, 2019, 2022.

The RRD between NTU and RR (Fig 6) indicates that from 2011 to 2022, the YREB exhibited the largest number of areas with lagging NTU disorders. The relative development status resembles an "inverted triangle": a majority of areas are under the category of "lagging NTU disorders," fewer areas show "co-limiting disorders," and the fewest areas exhibit "lagging RR disorders." In addition, the change in this relative development status over the study period is not significant, demonstrating a weak trend of synchronous development between NTU and RR. This finding suggests that the development pace of NTU in each city is slower compared to RR, there is a mismatch between the pace of urban and rural development.

At the city level, only Suzhou, Hefei, and Kunming are categorized as experiencing co-limiting disorders of NTU and RR. Shanghai, Ningbo, Chengdu, Qujing, and Zhaotong transitioned from the state of lagging NTU disorders to co-limiting disorders. Specifically, Lijiang and Yuxi transitioned from a state of co-limiting disorders to lagging RR disorders, indicating a regressive trend in RR development. This regression might be attributed to the urban-rural dual development model of "emphasizes cities and neglects countrysides," which leads to issues such as the uneven distribution of resources and low efficiency of agricultural production.

Regarding regional disparities, cities in the MR are all in the state of lagging NTU disorders (ratio of 100%). In contrast, most cities in the DR and UR are in the state of lagging RR disorders, with proportions of 80.49% and 78.13% respectively. This disparity highlights the expanding leading position of NTU in the DR and UR. Both "lagging NTU disorders" and "lagging RR disorders" are detrimental to the benign development of the system. Regions lagging in NTU should focus on improving the quality of urbanization; whereas, regions lagging in RR should concentrate on enhancing the level of RR construction. These efforts are essential to promoting the coupled and coordinated development of NTU and RR at a high level.

### 3.3 Spatio-temporal changes in the CCD between NTU and RR

**Fig 7:**
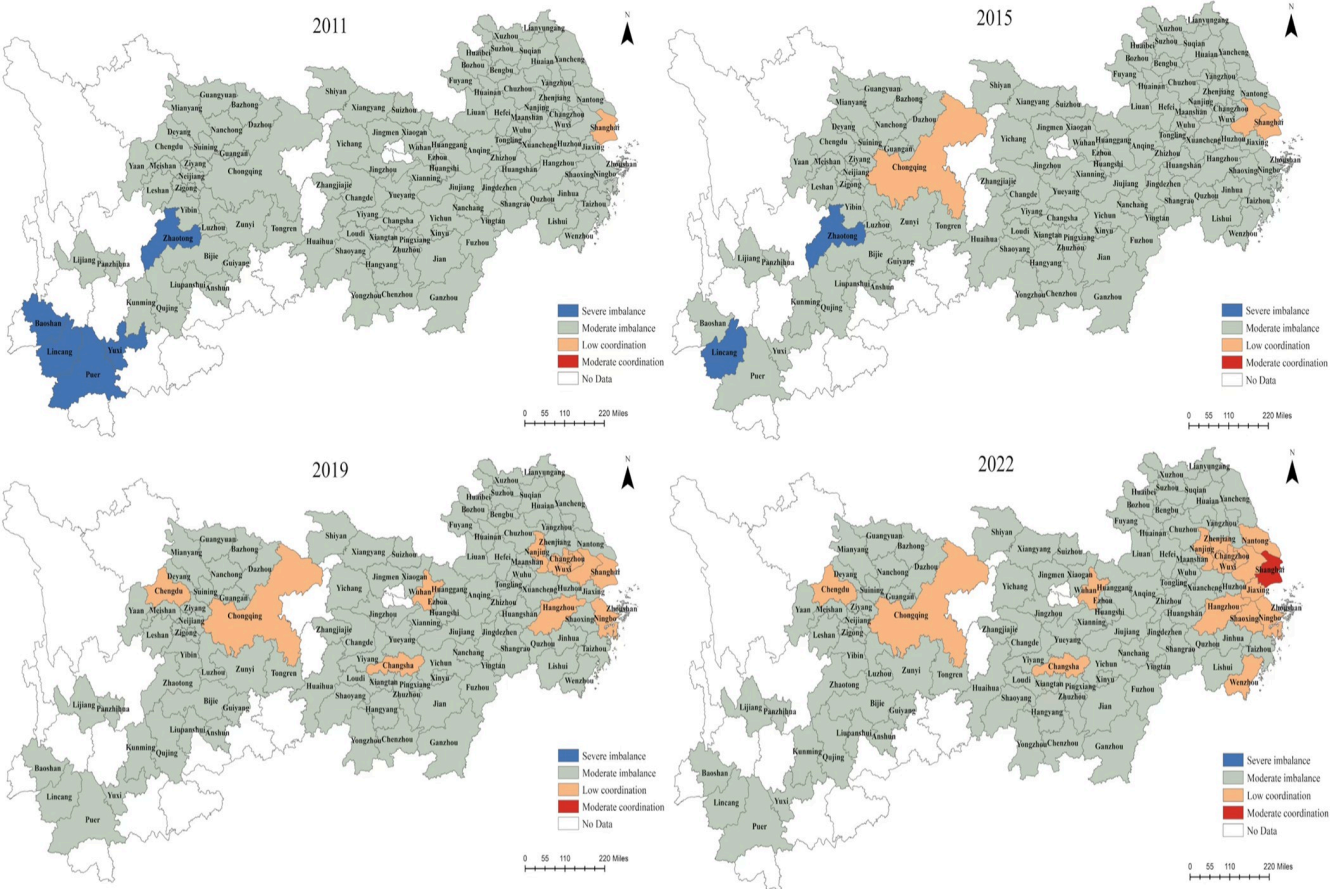
The CCD between NTU and RR, 2011, 2015, 2019, 2022.

The CCDM is employed to measure the CCD of NTU and RR in 110 cities in the YREB from 2011 to 2022, and a visualization diagram is presented (Fig 7). Findings indicate a relatively low CCD for both NTU and RR in the YREB during this period, but a trend of strengthening coordination between two systems is observed. The current type of moderate imbalance is expected to persist before transitioning to a state of primary coordination, showing a developmental progression from moderate imbalance towards low coordination.

Specifically, economically thriving cities such as Shanghai, Chongqing, Hangzhou, Suzhou, Nanjing, Wuhan, Ningbo, Wuxi, and Chengdu exhibit a higher CCD; whereas, economically challenged cities such as Dazhou, Ziyang, Bazhong, Ya’an, Lijiang, Yuxi, Qujing, Baoshan, Pu’er, Zhaotong, and Lincang demonstrate a lower CCD. This contrast highlights a significant difference in the coupled development level of NTU and RR across the YREB. The geographical distribution of the coupled coordination degree indicates a pattern: "DR > MR > UR, the interior of urban agglomerations > the exterior, and the central city> the surrounding cities." In addition, most cities in the UR and MR categories are consistently under the moderate imbalance stage. Only Chengdu, Chongqing, Changsha, and Wuhan have progressed from moderate imbalance to low coordination. The DR represents the high-value area for CCD, with the YRDUA, centered around Shanghai, displaying the highest values. However, a majority of cities in this area remain at the low coordination stage, indicating a positive trend of gradual growth in the number of cities transitioning to this category. This observation signifies a crucial period of transformation, shifting from dysfunctional states toward coordinated excesses.

### 3.4 Regional disparities of the CCD

**Fig 8:**
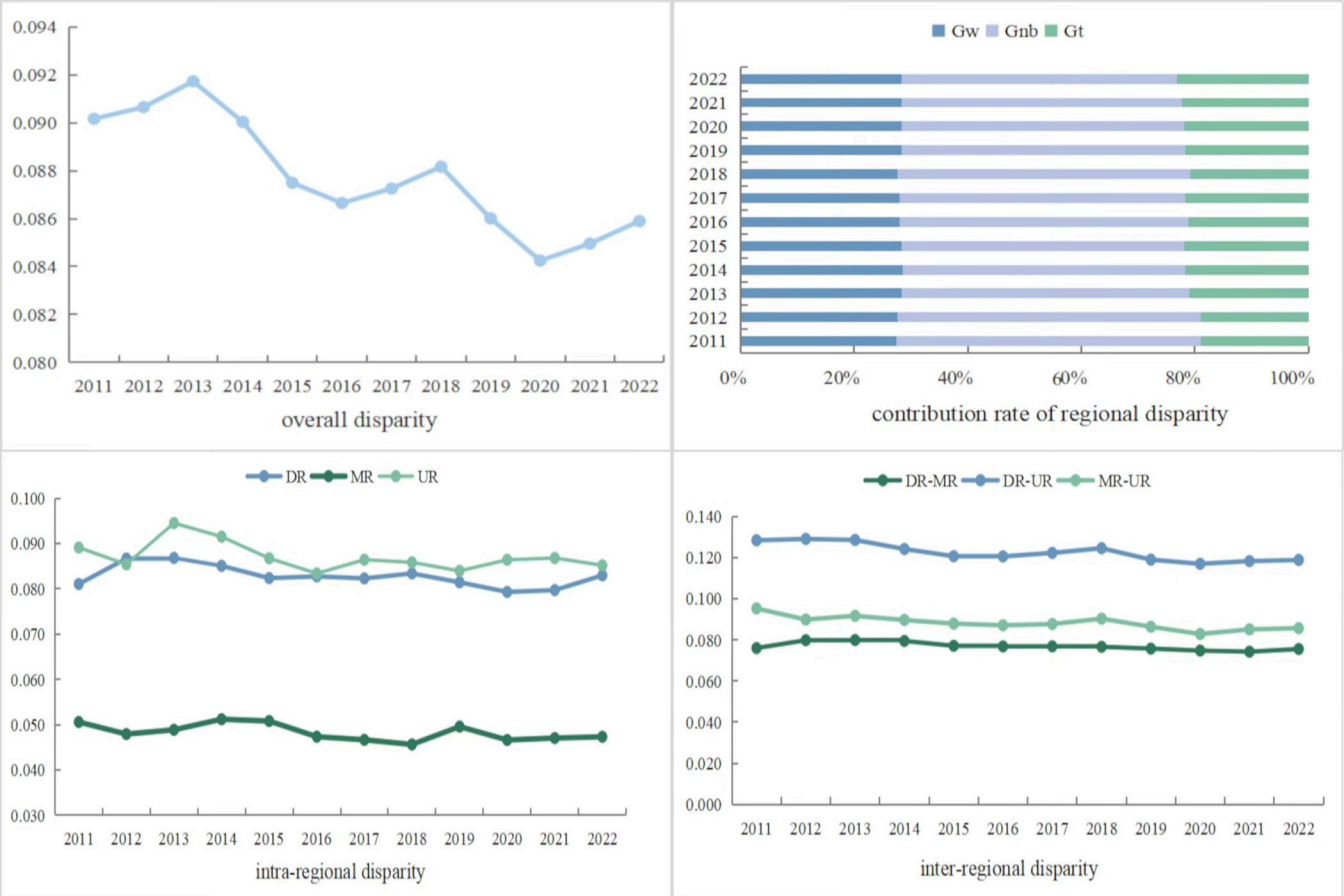
Regional differences of the CCD.

The regional disparities of the CCD between NTU and RR in the YREB are analyzed by the Dagum Gini coefficient (Fig 8).

In consideration of the overall disparity, we find that from 2011 to 2022, the overall Gini coefficient of CCD decreased, albeit with some fluctuations. The overall Dagum Gini coefficient exhibits an “M”-type trend reflected by a “rising-declining-rising-declining” pattern. It increased from 0.0902 (2011) to 0.0917 (2013), then began to decline to 0.0867 in 2016, subsequently grew by 0.8820 in 2018, then decreased again in 2019 and 2020, and thereafter grew by 0.0859 in 2022. This suggests a narrowing trend in the overall relative gap concerning the coupled development of NTU and RR in the YREB. The overall situation regarding the coupled development of NTU and RR appears to be improving, indicating that solutions aimed at addressing development imbalances have yielded some positive outcomes.

With respec to the source and contribution rate of regional disparity, the average contribution rates of intra-regional disparity, inter-regional disparity, and hypervariable density to the overall disparity of CCD in the YREB are 28.12%, 50.62%, and 21.26%, respectively. Specifically, inter-regional disparity represents the largest contributing factor, reaching 50%, significantly higher than both intra-regional disparity and hypervariable density. This highlights that intra-regional disparity constitutes the primary source of disparities in the coupled development level of NTU and RR in the YREB.

Focusing on intra-regional disparity, the trends observed in the intra-group disparity of CCD across the three major regions of the YREB (DR, MR, UR) are largely consistent. Each region exhibits a downward trend, while disparities exist in the magnitude of these values. Specifically, the mean value of the intra-group Gini coefficient follows the pattern of UR (0.0871) > DR (0.0828) > MR (0.0483) in the YREB. This suggests a tendency towards convergence in the unbalanced development of NTU and RR coupled between the UR and DR, while the disparity remains significantly larger in comparison to the MR. Nevertheless, the internal difference in CCD among the three regions has experienced a slight narrowing, indicating that these regions have made microscopic progress in promoting the coupled of NTU and RR. However, further efforts are required in this regard.

Regarding inter-regional disparity, a significant disparity persists in the CCD of NTU and RR among the three regions. Nonetheless, the overall trend remains consistent, demonstrating a gradual decline. The mean value of the Gini coefficient between groups is ranked from high to low as "DR—UR (0.1226) > MR—UR (0.0883) > DR—MR (0.0769) ", the Gini coefficient between the DR and UR decreased from 0.1284 to 0.1188, a year-on-year decrease of 7.45%; the Gini coefficient between the DR and the MR decreased from 0.0760 to 0.0755, a year-on-year decrease of 0.62%; the Gini coefficient between the MR and UR decreased from 0.0953 to 0.0856, a year-on-year decrease of 10.08%. These findings evidence that while significant inter-regional disparity remains, there is a clear trend towards a reduction in these disparities.

### 3.5 Dynamic evolution of the CCD

**Fig 9:**
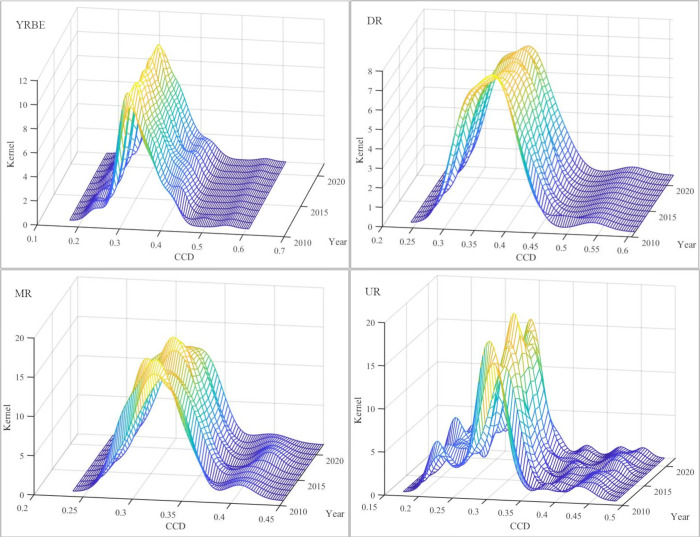
YREB’s kernel density curve of CCD.

To further explore the dynamic evolution characteristics of the CCD of NTU and RR, this study employed Matlab software to construct kernel density curves. These curves, representing the CCD of the YREB holistically, in addition to UR, MR, DR from 2011 to 2022, were plotted and analyzed across four aspects: distribution location, distribution pattern, distribution extensibility, and polarization characteristics (Fig 9).

From 2011 to 2022, the position of the main peak of the kernel density curve for the CCD in the YREB, including the UR, MR, and DR, generally demonstrated a rightward shift, with leftward shifts observed only in select years. This rightward shift suggests a gradual enhancement of the CCD of NTU and RR in the YREB. The occurrence of occasional leftward shifts during the studied period imply the existence of continuing constraints and challenges in promoting the coupled development of the YREB.

The kernel density curve of the YREB as a whole presented a "fluctuating decrease in the height of the main peak and a slight increase in the width of the main peak." This pattern signifies a widening absolute disparity in CCD across the YREB. The DR demonstrated a "decrease in the height of the main peak and increase in the width of the main peak," consistent with the MR The main peak height of the UR demonstrated a fluctuating change of "↑-↓-↑-↓-↑" and the width of "↓-↑-↓-↑-↓;" however, in general, it demonstrated a tendency of slightly decreasing in height and increasing in width. These patterns collectively suggest a widening absolute disparity in the coupled development of NTU and RR across all three regions.

The right trailing phenomenon of the kernel density curve in the YREB is evident, while the length of the right trailing exhibited a slight reduction. This observation indicates that the disparity between the cities with high degrees of coordination and the cities with low degrees of coordination in the YREB has reduced. In both the DR and MR, a right trailing phenomenon was observed, and the distribution extensibility demonstrated a trend of convergence. This finding implies that the growth rate of the CCD has declined in the cities with a higher level of coupled development and is approaching the average level of their respective regions; whereas, the UR did not exhibit a trailing phenomenon, indicating that a significant spatial disparity in the development of intra-regional coupling is essentially absent.

The kernel density curves of the YREB, DR, and MR each presented only one wave peak and did not display polarization characteristics. In contrast, the UR exhibited one main peak and one side peak in 2012, 2015, 2017, and 2021, while multiple wave peaks were observed in 2022. This suggests that the UR’s coupled development exhibits prominent polarization characteristics.

### 3.6 Influence factor of the CCD

Analysis employing a Tobit model yielded LR test results that strongly rejected the original hypothesis of "no individual effects," indicating that a panel Tobit model with random effects is appropriate for conducting regression analysis. Accordingly, regressions were performed on the complete YREB sample, along with the upstream, midstream, and downstream samples (Table 4), to appraise the factors (OPL, TL, TIL, FSAL, AML, and AID) that affect the CCD of NTU and RR, and to study how these influencing factors differ across the regions.

**Table 4:** Results of the analysis of factors affecting the CCD.

D	YREB	DR	MR	UR
OPL	0.003***(4.84)	0.003**(2.28)	0.008***(6.00)	0.001(0.99)
TL	0.008***(3.58)	0.002(0.70)	0.030***(5.18)	0.009**(2.46)
TIL	0.017***(25.39)	0.020***(14.17)	0.012***(11.73)	0.018***(15.80)
FSAL	-0.010***(-4.69)	-0.019***(-6.42)	0.005(1.49)	-0.001(-0.32)
AML	0.006**(2.37)	0.006(1.63)	0.004(1.23)	0.005(1.14)
AID	0.062***(16.28)	0.076***(10.70)	0.065***(9.78)	0.040***(6.24)
Constant	-0.445***(-15.86)	-0.515***(-10.83)	-0.733***(-12.67)	-0.287***(-6.29)
N	1,320	492	432	396
id	110	41	36	33

Note: ***, **, * indicate 1%, 5%, and 10% significance levels, respectively, with t-values in parentheses.

#### 3.6.1 A regression analysis conducted on the entire YREB sample

OPL demonstrates a regression coefficient of 0.003, exhibiting significant positivity at the 1% level. This can be attributed to the large investment space and potential in the YREB, attracting foreign investment for NTU construction and RR development. Such investments cultivate novel agricultural foreign partnerships, elevate the quality of agricultural trade, and facilitate the reciprocal exchange of agricultural science and technology cooperation. Therefore, this achieves to a broader scope, expanded fields, and a more significant level of agricultural openness to the outside world, with a significant driving effect on the coupled development of NTU and RR.

The regression coefficient of TL is 0.008, also significantly positive at the 1% level. This result is likely due to the accelerated development of a convenient, efficient, inclusive, and equitable rural road network, coupled with the creation of an integrated urban-rural transportation system. This effectively revitalizes both urban and rural transportation resources, and provides important support for the good interaction between NTU and RR.

TIL presents a regression coefficient of 0.017, significantly positive at the 1% level. This positive effect can be associated with improvements in the conversion rate of scientific and technological achievements. Promoting the efficiency of agricultural production, and becoming an endogenous driving force to promote the coupled development of NTU and RR.

The regression coefficient of AML stands at 0.006, significantly positive at the 5% level. This result may be attributed to the comprehensive advancement of structural reforms in the agricultural mechanization supply side. The acceleration of agricultural mechanization towards a comprehensive, high-quality, and high-efficiency model enables economies of scale in agricultural production, and generating a scaling effect that furthers the coupled development of NTU and RR.

AID exhibits a regression coefficient of 0.062, significantly positive at the 1% level, demonstrating the most significant driving force behind the coupled development of NTU and RR in the YREB. This significant effect may be attributed to the emphasis placed on the development of the agricultural product processing industry. Therefore, it represents an essential choice for advancing the coupled development of NTU and RR.

Whereas, FSAL presents a regression coefficient of -0.010, significantly negative at the 1% level. This negativity potentially arises from a degree of "weight over quality" and "hard over soft" tendencies observed in the allocation of agriculture-related funds across various levels of government. This misalignment with the genuine needs of urban and rural construction impacts the effective utilization of financial funds. Therefore, greater emphasis should be directed towards enhancing the efficiency of financial support for agriculture to fully leverage its positive effect on the coupled development of NTU and RR.

#### 3.6.2 A regression analysis conducted on the subregional level

TIL (positive at a 1% significance level), AID (positive at a 1% significance level), and AML (positive but statistically insignificant regression coefficients) exert similar effects on the CCD of NTU and RR in the UR, MR, and DR. However, the DR exhibits the largest coefficient value, suggesting that TIL, AID, and AML exert more significant effects on the CCD of NTU and RR in this region. This finding may be attributed to the higher levels of TIL, AID, and AML observed in the DR compared to the MR and UR, offering a more robust basis for the coupled development of NTU and RR.

The roles of OPL and TL in influencing the CCD of NTU and RR differ slightly across the UR, MR, and DR. The DR’s OPL (regression coefficient of 0.003, P<0.05) significantly and positively affects CCD, while TL (regression coefficient of 0.002, insignificant P-value) does not demonstrate a significant effect. This difference may be as the DR, represented by its early adoption of open-door policies, established foundation, rapid development, and relatively high level of development, effectively promotes the coupled development of NTU and RR. While transportation infrastructure is advanced, it did not make a significant contribution. Whereas, both the MR’s OPL (regression coefficient of 0.008, P<0.01) and TL (regression coefficient of 0.030, P<0.01) have a significant positive effect on CCD. The coefficient values are higher than those observed in the UR and DR, indicating a stronger promotional effect of OPL and TL on the coupled development of NTU and RR in the MR compared to the UR and DR.The emergence of inland open hubs in the MR, represented by Wuhan, Changsha, and Nanchang, may be contributing to this phenomenon. These hubs demonstrate clear openness advantages and enhanced transportation capacity, which significantly strengthen the coupled development of NTU and RR. The insignificant effect of OPL in the UR (regression coefficient of 0.001, P value is insignificant) contrasts with the significant positive effect of TL (regression coefficient of 0.009, P<0.05) on CCD. This suggests that robust transportation is crucial for the coupled development of NTU and RR. This contrast may arise because the positive effects of OPL have not yet fully materialized. Meanwhile, accelerated development of new land and sea corridors in the west has improved transportation in the UR. This improvement has facilitated the two-way flow of resources between urban and rural areas, significantly and positively affecting CCD.

The effect of FSAL on the CCD of NTU and RR across the UR, MR, and DR is heterogeneous. While FSAL negatively affects the DR (regression coefficient is -0.019, P<0.01), it demonstrates no significant effect on the UR (regression coefficient is -0.001, P value is not significant). This difference likely arises as comprehensively promoting RR necessitates optimizing the use of agriculture-related funds. However, the efficiency of this fund utilization remains suboptimal, and the management system needs improvement, finally hindering the coupled development of NTU and RR; whereas, FSAL demonstrates a positive effect on the MR (regression coefficient of 0.05, P value is not significant). This positive effect may be attributed to increased financial support for agricultural inputs, which offers a foundation for urbanization and rural development. However, the current level of support is insufficient to significantly boost the coupled development of NTU and RR.

Overal, in any of the DR, or MR and UR, OPL, TL, TIL, AML, and AID positively contribute to the coupled development of NTU and RR. However, FSAL demonstrates a positive effect in the MR and a negative effect on both the UR and DR. In addition, AID has the most significant effect on the three regions, followed by TIL.

## 4. Conclusions and discussion

This paper, leveraging Stata, Matlab, and ArcGIS software, employs entropy methods, RDDM, CCDM, Dagum Gini coefficient, Kernel density estimation, and Tobit model to explore the spatial-temporal changes, regional disparities, and influencing factors of the coupled development of NTU and RR in the YREB from 2011 to 2022. The main conclusions of the study are as follows:

(1) The NTU and RR indices indicates consistent yearly growth for both in the YREB, with the majority of cities featuring a comparatively low developmental level. Furthermore, the spatial distribution is uneven, exhibiting significant regional disparities. Notably, the relative development status resembles an "inverted triangle": a majority of areas are under the category of "lagging NTU disorders," fewer areas show "co-limiting disorders," and the fewest areas exhibit "lagging RR disorders".

(2) The coupled development of NTU and RR in the YREB is insufficient and unbalanced, and most of the cities have a low CCD, which belongs to the type of moderate imbalance. The geographical distribution pattern adheres to the hierarchy of "DR > MR > UR, the interior of urban agglomerations > the exterior, and the central city> the surrounding cities".

(3) The relative disparity in the coupled development of NTU and RR in the YREB demonstrates a narrowing trend. The overall, intra-group, and inter-group Gini coefficient exhibit a general decline amidst occasional fluctuations, with intra-regional differences being the main source of their regional disparities.

(4) The absolute disparity in the coupled development of NTU and RR in the YREB demonstrates a expanding tendency. The kernel density curves of the CCD across the YREB and its three regions indicate a rightward shift in the main peak, a decrease in peak height, and an increase in peak width, yet their distribution extensibility and polarization characteristics traits vary.

(5) The influencing factors (OPL, TL, TIL, AML, and AID) have a positive promoting effect on the coupled development of NTU and RR in the YREB, of which AID has the strongest driving effect. Conversely, FSAL has a negative inhibitory effect, which is heterogeneous in nature.

Based on these findings, the following countermeasures are proposed:

(1) The majority of the cities belonging to the lagging NTU disorders should expedite a human-centric approach to NTU development. Some cities are experiencing a deceleration in RR, it is essential to address deficiencies, strengthen foundations, and encourage reform and innovation. Nurture and invigorate novel sources of momentum and advantages within RR, amplify its developmental impetus, and make efforts to reduce the disparity between the urban and rural development within the YREB.

(2) In the DR, sustaining the rapid improvement of coupled coordination between NTU and RR should be coupled with enhancing its radiation-driven role. This can be achieved by improving the institutional mechanism for managing agricultural financial funds, strengthening the connection between opening to the outside world and the transformation of scientific and technological achievements, and accelerating the development of the agricultural product processing industry. Actively exploring coupled mechanisms to better synchronize NTU and RR is crucial for realizing deep urban-rural integration. The MR should prioritize developing the agricultural product processing industry while synergistically promoting opening-up, transportation, and scientific and technological innovation. It should also leverage financial support for agriculture and agricultural mechanization to fully realize their positive effects on NTU-RR coupled. The UR must prioritize leveraging transportation, scientific and technological innovation, and the development level of the agricultural product processing industry to promote the coupled development of NTU and RR. This will enable it to accelerate coupled development, narrow regional regional disparities with the DR and MR, and enhance the regional balance of NTU-RR coupled development.

(3) On the one hand, it is necessary to vigorously develop the agricultural product processing industry and cultivate leading enterprises. This facilitates the transformation of agricultural product processing from primary to deep and intensive processing, finally prolonging the agricultural product industrial chain. This effectively promotes the coupled development of NTU and RR in the YREB. On the other hand, it is more important to improve the efficiency of agriculture-related funds. This involves guiding and leveraging financial capital into NTU and RR construction. We should strive to build a financial support system for agricultural policies that aligns with their coupled development. This allows for the full realization of the multiplier effect of financial funds.

This study still has some limitations. Firstly, this paper builds a comprehensive indicator system for NTU and RR by selecting important indicators based on existing research, the system cannot fully express the two systems’ connotations due to limitations in data acquisition at the prefecture-level city level. Further research should gain greater insights to connotations of NTU and RR scientifically, which may pave the pathway to a more comprehensive exploration of their evaluation indicator and influencing factor system. Secondly, the paper omits a qualitative analysis of the current conditions of NTU and RR, as well as their coupled development, in the YREB. Future research should clarify the challenges and limitations related to this coupling from a qualitative perspective to establish a more comprehensive theoretical foundation. In addition, further exploration of spatial effects is necessary as this paper does not involve the study of spatial effect. Future research should consider employing methods such as spatial autocorrelation analysis and panel spatial econometric models to enhance the research framework, and contribute to a more comprehensive understanding of the spatial effect of the coupled development of NTU and RR of the YREB.

## Supporting information

S1 Data (XLSX)

## References

[1] Yao Y., Jiang L. Urbanization forces driving rural urban income disparity: Evidence from metropolitan areas in China. Journal of Cleaner Production.2021; 312, 127748. 10.1016/j.jclepro.2021.127748

[2] Lin B., Zhu J. Impact of China’s new-type urbanization on energy intensity: A city-level analysis. Energy Economics.2021; 99, 105292. 10.1016/j.eneco.2021.105292

[3] Cui H., Cao Y. China’s cities go carbon neutral: How can new-type urbanization policies improve urban carbon performance? Sustainable Production and Consumption.2023; 42, 74–94. 10.1016/j.spc.2023.09.011

[4] Chen C., Qin Y., Gao Y. Does new urbanization affect CO2 emissions in China:A spatial econometric analysis. Sustainable Cities and Society.2023; 96, 104687. 10.1016/j.scs.2023.104687

[5] Zhang Q., Kong Q., Zhang M., Huang H. New-type urbanization and ecological well-being performance: A coupling coordination analysis in the middle reaches of the Yangtze River urban agglomerations, China. Ecological Indicators.2024; 159, 111678. 10.1016/j.ecolind.2024.111678

[6] Yu B. Ecological effects of new-type urbanization in China. Renewable and Sustainable Energy Reviews.2021; 135, 110239. 10.1016/j.rser.2020.110239

[7] Chen M., Liu W., Lu D., Chen H., Ye C. Progress of China’s new-type urbanization construction since 2014: A preliminary assessment. Cities.2018; 78, 180–193. 10.1016/j.cities.2018.02.012

[8] Ma L., Xiang L., Wang C., Chen N., Wang W. Spatiotemporal evolution of urban carbon balance and its response to new-type urbanization: A case of the middle reaches of the Yangtze River Urban Agglomerations, China. Journal of Cleaner Production.2022; 380, 135122. 10.1016/j.jclepro.2022.135122

[9] Tu D., Cai Y., Liu M. Coupling coordination analysis and spatiotemporal heterogeneity between ecosystem services and new-type urbanization: A case study of the Yangtze River Economic Belt in China. Ecological Indicators.2023; 154, 110535. 10.1016/j.ecolind.2023.110535

[10] Chen M., Gong Y., Lu D., Ye C. Build a people-oriented urbanization: China’s new-type urbanization dream and Anhui model. Land Use Policy.2019; 80, 1–9. 10.1016/j.landusepol.2018.09.031

[11] Deng S. Exploring the relationship between new-type urbanization and sustainable urban land use: Evidence from prefecture-level cities in China. Sustainable Computing: Informatics and Systems.2021; 30, 100446. 10.1016/j.suscom.2020.100446

[12] Zhang W., Xu Y., Streets D. G., Wang C. Can new-type urbanization realize low-carbon development? A spatiotemporal heterogeneous analysis in 288 cities and 18 urban agglomerations in China. Journal of Cleaner Production.2023; 420, 138426. 10.1016/j.jclepro.2023.138426

[13] Wu Y., Zhou C., Lai X., Li Y., Miao L., Yu H. Spatio-temporal characteristics and decoupling relationship of new-type urbanization and carbon emissions at the county Level: A case study of Zhejiang Province, China. Ecological Indicators.2024; 160, 111793. 10.1016/j.ecolind.2024.111793

[14] Zhang M., Tan S., Zhang Y., He J., & Ni Q. Does land transfer promote the development of new-type urbanization? New evidence from urban agglomerations in the middle reaches of the Yangtze River. Ecological Indicators.2022; 136, 108705. 10.1016/j.ecolind.2022.108705

[15] Chen D., Hu W., Li Y., Zhang C., Lu X., Cheng H. Exploring the temporal and spatial effects of city size on regional economic integration: Evidence from the Yangtze River Economic Belt in China. Land Use Policy. 2023; 132, 106770. 10.1016/j.landusepol.2023.106770

[16] Zhang W., Wang M. Y. Spatial-temporal characteristics and determinants of land urbanization quality in China: Evidence from 285 prefecture-level cities. Sustainable Cities and Society.2018; 38, 70–79. 10.1016/j.scs.2017.12.011

[17] Chen M., Liu W., Lu D. Challenges and the way forward in China’s new-type urbanization. Land Use Policy. 2016; 55, 334–339. 10.1016/j.landusepol.2015.07.025

[18] Zhou D., Hu Y., Sun Q., Xie D. Land resource mismatch and energy efficiency: Evidence from 243 cities in China. Energy Policy.2023; 183, 113800. 10.1016/j.enpol.2023.113800

[19] Guan X., Wei H., Lu S., Dai Q., Su H. Assessment on the urbanization strategy in China: Achievements, challenges and reflections. Habitat International.2018; 71, 97–109. 10.1016/j.habitatint.2017.11.009

[20] Dwyer J., & Powell J. Rural development programmes and transaction effects: Reflections on Maltese and English experience. Journal of Agricultural Economics.2016; 67 (3), 545–565. 10.1111/1477-9552. 12166

[21] Noble V. Mobilities of the one-Product policy from Japan to Thailand: A critical policy study of OVOP and OTOP. Territory Politics Governance.2019; 7(4), 455–473. 10.1080/21622671.2018.1511463

[22] Li W, Zhang L, Lee I, Gkartzios M. Overview of Social Policies for Town and Village Development in Response to Rural Shrinkage in East Asia: The Cases of Japan, South Korea and China. Sustainability 2023;15(14), 10.3390/su151410781

[23] Joanne W. Is it time to revisit "Rural Proofing" of all Australian governments’ policy development and implementation? The Australian journal of rural health.2019; 27(2):188–189. doi: 10.1111/ajr.12514 31006951

[24] Hirschi C. Strengthening regional cohesion: Collaborative networks and sustainable development in Swiss rural areas. Ecology and Society.2010; 115(4), 16. 10.5751/ES-03714-15041

[25] Xi J. The rural revitalization strategy: the key to our efforts concerning agriculture, rural areas and farmers in the New Era. Qiushi 2–5. www.qstheory.cn/ dukan/qs/2019-06/01/c_1124561415.htm

[26] Zhao Q., Bao H. X. H., Yao S. Unpacking the effects of rural homestead development rights reform on rural revitalization in China. Journal of Rural Studies.2024; 108, 103265. 10.1016/j.jrurstud.2024.103265

[27] Xu Q., Zhong M., Dong Y. Digital finance and rural revitalization: Empirical test and mechanism discussion. Technological Forecasting and Social Change.2024; 201, 123248. 10.1016/j.techfore.2024.123248

[28] Wang J., 2023. Digital inclusive finance and rural revitalization. Finance Research Letters. 57, 104157. 10.1016/j.frl.2023.104157

[29] Deng X., Huang M., Peng R. The impact of digital economy on rural revitalization: Evidence from Guangdong, China. Heliyon. 2024; 10(7), e28216. doi: 10.1016/j.heliyon.2024.e28216 38601566 PMC11004695

[30] Xiong Z., Huang Y., Yang L. Rural revitalization in China: Measurement indicators, regional differences and dynamic evolution. Heliyon.2024; 10(8), e29880. doi: 10.1016/j.heliyon.2024.e29880 38699725 PMC11063445

[31] Geng Y., Liu L., Chen L. Rural revitalization of China: A new framework, measurement and forecast. Socio-Economic Planning Sciences.2023; 89, 101696. 10.1016/j.seps.2023.101696

[32] Li G., Zhang X. The Spatial–Temporal Characteristics and Driving Forces of the Coupled and Coordinated Development between New Urbanization and Rural Revitalization. Sustainability.2023; 15,16487. 10.3390/su152316487

[33] Yin J.F., Shi P.J., Huang W.Z., Shi Z.H., Li Y.L. Spatiotemporal differentiation characteristics and influencing factors of coupling coordinated development of rural revitalization and new urbanization at the county level in Gansu province. Journal of Natural Resources.2023; 38 (8), 2148–2168. 10.31497/zrzyxb.20230814.

[34] Cheng M, Fang Q. Research on the Coupling Mechanism of Rural Revitalization and New Urbanization Strategy: From the Perspective of Urban-Rural Factor Flow. East China Economic Management.2023; 37(05):1–8. 10.19629/j.cnki.34-1014/f.221014017.

[35] Zhang M. Strategic coupling and collaborative path of rural revitalization and new urbanization. Journal of Huazhong Agricultural University(Social Sciences Edition). 2022; (01):45–52. 10.13300/j.cnki.hnwkxb.2022.01.005.

[36] Weng F. Integration of Rural Revitalization Strategy and New Urbanization Construction: Experiences, Obstacles and New Era Programs. Dongyue Tribune.2020; 41(05):70–77. 10.15981/j.cnki.dongyueluncong.2020.05.028.

[37] Qiao G, Wang L, Du P. Contradiction or harmony? Spatial and temporal relationships between new urbanization and rural revitalization in the Yellow River Basin from a coupling perspective. PLoS ONE. 2023; 18(7): e0288600. doi: 10.1371/journal.pone.0288600 37471319 PMC10358882

[38] Chen M., Zhou Y., Huang X., Ye C. The Integration of New-Type Urbanization and Rural Revitalization Strategies in China: Origin, Reality and Future Trends. Land.2021; 10(2), 207; 10.3390/land10020207

[39] Sui Y., Hu J., Zhang N., Ma F. Exploring the dynamic equilibrium relationship between urbanization and ecological environment—A case study of Shandong Province, China. Ecological Indicators,2024; 158, 111456. 10.1016/j.ecolind.2023.111456

[40] He B., Du X., Lu Y., Chen Q., Lan R. An improved approach for measuring the coupling relationship between new type urbanization and low carbon development in China. Ecological Indicators.2024; 158, 111383. 10.1016/j.ecolind.2023.111383

[41] Yin Q.Q., Sui X.Y., Ye B., Zhou Y.J., Li C.Q., Zou M.M., et al. What role does land consolidation play in the multi-dimensional rural revitalization in China? A research synthesis. Land Use Policy.2022; 120, 106261. 10.1016/j.landusepol.2022.106261

[42] Shao J., Zhang L., Cai C. Dynamic evolution and spatial spillover effect of agricultural green development on eight economic regions in China. Heliyon.2024; 10(12), e33188. doi: 10.1016/j.heliyon.2024.e33188 39005913 PMC11239686

[43] Long X., Wu S., Wang J., Wu P., Wang Z., Urban water environment carrying capacity based on VPOSR-coefficient of variation-grey correlation model: a case of Beijing, China, Ecol. Indic. 2022;138 108863, 10.1016/j. ecolind.2022.108863.

[44] Gisleine C.Z., Ferreira G.J., Albiach B.E., Pierre O.J., How sustainable is the nitrogen management in Brazil? A sustainability assessment using the Entropy Weight Method, J. Environ. Manag. 2022;316, 115330, https://doi.org/10.1016/ j.jenvman.2022.115330. 10.1016/j.jenvman.2022.115330 35658265

[45] Hu Z., Kumar J., Qin Q., Kannan S. Assessing the coupling coordination degree between all-for-one tourism and ecological civilization; case of Guizhou, China. Environmental and Sustainability Indicators.2023; 19, 100272. 10.1016/j.indic.2023.100272

[46] Zou C., Zhu J., Lou K., Yang L. Coupling coordination and spatiotemporal heterogeneity between urbanization and ecological environment in Shaanxi Province, China. Ecological Indicators.2022; 141, 109152. 10.1016/j.ecolind.2022.109152

